# Watershed Brain Regions for Characterizing Brand Equity-Related Mental Processes

**DOI:** 10.3390/brainsci11121619

**Published:** 2021-12-08

**Authors:** Shinya Watanuki

**Affiliations:** Department of Marketing, Faculty of Commerce, University of Marketing and Distribution Sciences, Kobe 651-2188, Japan; Shinya_Watanuki@red.umds.ac.jp; Tel.: +81-(0)78-796-2543

**Keywords:** consumer neuroscience, ALE, AutoML, brand management, episodic memory, brand association

## Abstract

Brand equity is an important intangible for enterprises. As one advantage, products with brand equity can increase revenue, compared with those without such equity. However, unlike tangibles, it is difficult for enterprises to manage brand equity because it exists within consumers’ minds. Although, over the past two decades, numerous consumer neuroscience studies have revealed the brain regions related to brand equity, the identification of unique brain regions related to such equity is still controversial. Therefore, this study identifies the unique brain regions related to brand equity and assesses the mental processes derived from these regions. For this purpose, three analysis methods (i.e., the quantitative meta-analysis, chi-square tests, and machine learning) were conducted. The data were collected in accordance with the general procedures of a qualitative meta-analysis. In total, 65 studies (1412 foci) investigating branded objects with brand equity and unbranded objects without brand equity were examined, whereas the neural systems involved for these two brain regions were contrasted. According to the results, the parahippocampal gyrus and the lingual gyrus were unique brand equity-related brain regions, whereas automatic mental processes based on emotional associative memories derived from these regions were characteristic mental processes that discriminate branded from unbranded objects.

## 1. Introduction

Generally, branded products with brand equity are traded at premium prices, compared with unbranded products [1]. As for brand equity, it is one of the crucial profitable sources for enterprises and one of their greatest assets. Aaker [2] segregated the elements of brand equity into five factors: brand name awareness, brand associations, brand loyalty, perceived brand quality, and other proprietary brand assets, such as patents, trademarks, and channel relationships. Thus, unlike factories and office buildings, brand equity is an asset attributed to consumers’ minds.

Numerous brand equity studies have been conducted over the past two decades in order to clarify how to build brand equity in consumers’ minds. Such research included empirical studies, theoretical studies, and practical cases. Meanwhile, the number of neuroscience studies to understand brand equity-related mental processes has been increasing. For example, McClure et al. [3] showed that brain activations on beverage products with brand equity were observed in the hippocampus (HP) and dorsolateral prefrontal cortex (DLPFC), whereas both the ventral medial prefrontal cortex (VMPFC) and ventral striatum (VS) were activated in low-brand equity products. However, the activations of both the VMPFC and VS were observed in several brand equity studies [4,5,6,7,8]. These regions are known as the “neural currency network” [9]. The activations in these regions were also reported in several studies on unbranded objects [10,11,12].

Barring these regions, the activations in the medial prefrontal cortex (MPFC) were observed in both branded and unbranded research. For example, in a comparison between familiar automobile brands and unfamiliar ones, the MPFC was activated [13]. In related studies, Schaefer and Rotte [14] confirmed the activations in the MPFC when comparing luxury automobile brands and unfamiliar brands, whereas Chen et al. [15] investigated the brain activations associated with brand personality, which is an element of brand association composed of brand equity. In the latter study, they reported the activations in the MPFC, the cingulate cortex, and the caudate. Meanwhile, the activations in the MPFC were reported in numerous studies on unbranded objects [16,17,18,19,20,21,22,23,24,25,26,27]. Besides these brain regions, other brain activated regions have been found in studies on branded objects with brand equity, e.g., the insula [28], the inferior frontal gyrus [29], and the superior frontal gyrus [30,31]. It has also been reported that these brain regions are solely or multi-regionally activated.

Based on the aforementioned findings, brand equity-related brain regions are highly diversified. Even though brand equity has an influence on consumers’ decision-making, such as purchases, preferences, and attitudes, the watershed brain regions between brand equity and unbranded-related brain regions remain unknown. Therefore, the purpose of this study is to assess the unique characteristics of the mental processes associated with brand equity by identifying the watershed brain regions through a comparison between the brain regions related to brand equity and such regions related to unbranded objects without brand equity.

## 2. Materials and Methods

In order to achieve our research objective, the analyses were conducted in two steps. First, an activation likelihood estimation (ALE) was conducted to statistically clarify the distinct brain regions between branded objects with brand equity and unbranded objects without brand equity. Second, although a statistical conjunction analysis was attempted (based on the ALE method) to identify any overlapped or distinct brain regions between the brand equity-related brain regions and the unbranded objects-related brain regions, it could not be executed. Thus, a chi-square test was adopted to characterize the brand equity-related brain regions and the unbranded ones. When conducting the chi-square test, all the reported foci were categorized into the optimal number of clusters using the k-means algorithm. To assess the brain regions that can discriminate between brain equity-related brain regions and unbranded objects-related brain regions, supervised machine learning was applied. The details regarding these procedures are presented in the following sections.

### 2.1. Procedures of the ALE Method

A systematic literature review was conducted to select the neuroimaging studies on consumers’ decision-making of branded and unbranded objects. The selections were performed using PubMed (https://pubmed.ncbi.nlm.nih.gov) as the primary database. Specifically, the search focused on studies using functional magnetic resonance imaging (fMRI), with specific terms such as “brand”, “consumer”, “fMRI”, “neural”, “choice”, “purchase”, “decision-making”, and “preference”. This search process yielded the following: 10 studies for “brand, fMRI, neural, and choice”; 0 for “brand, fMRI, neural, and purchase”; 11 for “brand, fMRI, neural, and decision-making”; 12 for “brand, fMRI, neural, and preference”; 38 for “consumer, fMRI, neural, and choice”; 12 for “consumer, fMRI, neural, and purchase”; 48 for “consumer, fMRI, neural, and decision-making”; and 26 for “consumer, fMRI, neural, and preference”.

Next, the branding studies in Plassmann’s [32] references list were added. Based on the information in the titles and abstracts, the studies were selected according to the following criteria: (1) studies in peer-reviewed English language journals published between January 2000 and March 2021; (2) studies that conducted fMRI scans of healthy participants; (3) studies in which branded objects were used as experimental stimuli, e.g., products, logos, and advertising with brand logos or the equivalent; (4) studies in which unbranded objects were used as experimental stimuli, e.g., products, product packages, and advertisements without brand logos or the equivalent; and (5) studies that reported activations as three-dimensional coordinates in the stereotactic space of Talairach or the Montreal Neurological Institute (MNI).

**Figure 1:**
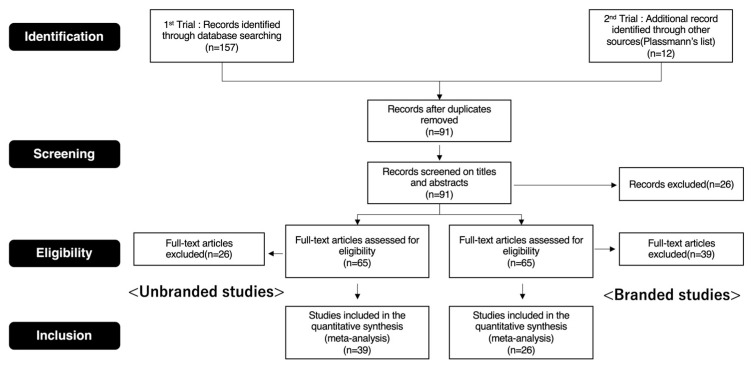
Prisma flow diagram.

It should be noted that two studies [33,34] did not directly use branded objects as experimental stimuli. However, they were ultimately included in the branded objects group for quantitative synthesis because the stimuli were regarded as objects similar to branded ones. These two studies were also included in Plassmann’s references list [32]. In addition, since Knutson et al. [35] adopted a shop task as an experimental task, it is believed that the stimuli used in their study included logos and characteristic package designs. However, their study was included in the unbranded objects group because they controlled the attractiveness of the stimuli, and their objectives assessed the influences of brand equity on consumers’ decision-making. In other words, they treated all the experimental stimuli as equivalents. The preferred items for the systematic review and meta-analyses (PRISMA) flow diagram (see Figure 1) provide details of this screening process. For the present meta-analysis (see Supplementary Table S1a,b), 26 studies (679 foci) were included in the branded objects group, while 39 studies (733 foci) were included in the unbranded objects group. In addition, similar to other meta-analytical neuroimaging studies, the entire activation foci of the included studies that were originally reported in the Talairach space were converted to the MNI space using a transformation algorithm [36], owing to disparities between the Talairach space and MNI space [37]. The MNI coordinates were adopted in the current study as an ALE map was created on the MNI space by using the GingerALE software (http://www.brainmap.org/, accessed on 1 April 2021) [38,39]. The details of the ALE method are described in Figure 1.

The ALE method [40], the most popular qualitative meta-analysis method [41], was also adopted. First, the modeled activation map was created by applying the three-dimensional Gaussian probability density function to each focus. Similar procedures were conducted on all the foci in the selected studies. With the increased convergence of the reported foci across studies, there was a gradual minimization in the variance of the Gaussian probability distribution. This indicated that the contingency of the reported foci in each study was expected to be eliminated. Second, an ALE map was obtained by calculating the union of the modeled activation maps. Finally, to create a more accurate ALE map, it was compared with the randomness map created by null distribution. Concretely, the ALE map with thresholds was obtained by conducting a permutation test, which assessed the differentiations between these maps at each voxel [40,42].

Overall, the ALE method was conducted using the GingerALE (Version 3.02) tool (http://www.brainmap.org/, accessed on 1 April 2021), while the thresholding analyses were performed using a cluster-level correction for multiple comparisons at *p* = 0.05, with a cluster-forming threshold of *p* = 0.0001. Meanwhile, the permutation size was set to 1000. Although Eickhoff et al. [43] recommended a cluster-forming threshold at *p* = 0.001, more conservative criteria were adopted. In the present study, all the coordinates were reported in the MNI space. Moreover, all the activated brain images were exported as NIfTI files and as output into the canonical anatomical T1 brain template in the MNI space via Mango software (Version 4.1; http://ric.uthscsa.edu/mango/, accessed on 1 April 2021).

### 2.2. Procedures of the Chi-Square Test

**Figure 2:**
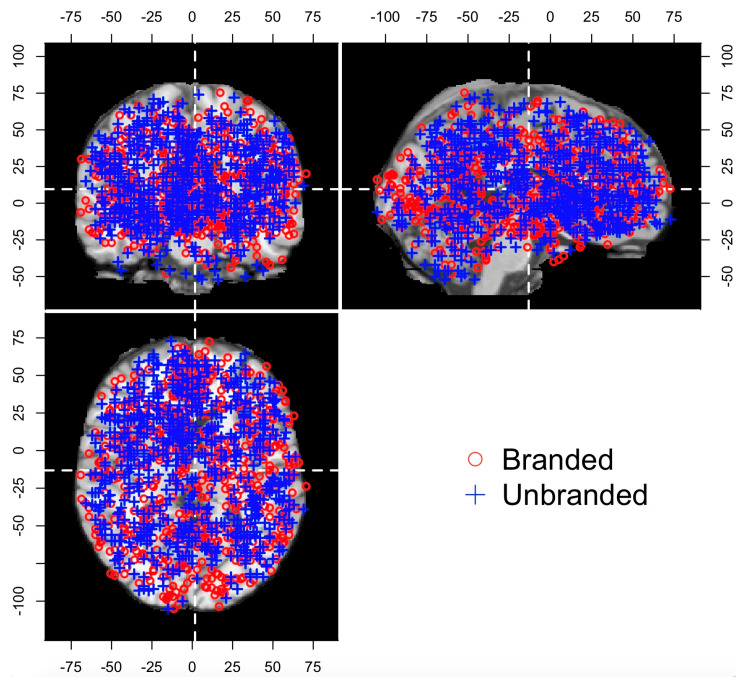
All foci plotted on the Colin27 template. Top left = coronal view; Top right = sagittal view; Lower right = axial view.

**Figure 3:**
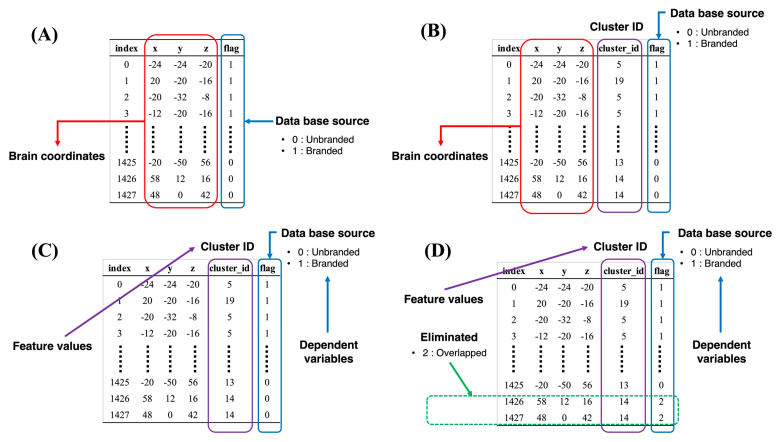
Explanations of the data structure. (**A**) Initial data structure. (**B**) Data structure after k-means clustering. (**C**) Data structure for machine learning. (**D**) Overall explanation of feature engineering.

The procedures of the chi-square test are as follows: First, the foci of both the branded studies and the unbranded studies were merged. Second, each focus was flagged, depending on each study’s database. Specifically, the foci in the branded studies’ databases were flagged as “1” (hereafter called the branded flag), since these foci were collected to measure the objects with brand equity. Meanwhile, the foci in the unbranded studies’ databases were flagged as “0” (hereafter called the unbranded flag), since these foci were collected to measure the unbranded objects. These data were also constructed from 1412 rows and four columns (excluding the index column). Each row represented each focus, while the first three columns (excluding the index column) represented the brain coordinates. The final column was expressed as the database source flags that indicated whether the foci corresponded to the branded or unbranded objects. These foci plotted on the Colin27 template are displayed in Figure 2, the details of the data structure are presented in Figure 3A, and the descriptive statistics are shown in Table 1. Third, in order to categorize each focus, the spatially closer brain coordinates were organized into appropriate clusters by using the k-means algorithm (see Figure 3B). In this case, the optimal number of clusters was determined though the elbow method, while the k-means algorithm was performed by using scikit-learn in Python. After the optimal number of clusters was determined and a cluster ID was provided to each focus, a chi-square test was conducted with both the cluster ID and the flag in order to determine whether each cluster included such tendencies as brand equity-related brain regions, unbranded-related brain regions, and overlapped brain regions across brand equity-related brain regions and unbranded-related brain regions (hereafter called overlapped brain regions).

### 2.3. Procedures of Machine Learning

The cluster ID, calculated in previous procedures, was used as a feature value, while the last column was used as a dependent variable. The data structure for machine learning is shown in Figure 3C. First, feature engineering was conducted to determine the values that have high relevance to the dependent variables. In this regard, if the cluster ID did not significantly differ via the results of the chi-square test, then the foci with the cluster ID were judged as overlapped brain regions, flagged as “2” (see Figure 3D), and eliminated. Second, supervised learning algorithms were performed to identify the brain regions. The calculations were performed using H2O-AutoML (H2O Version 3.32.1.2, https://www.h2o.ai, accessed on 15 July 2021), which is an open-source framework for machine learning [44]. Several major machine learning algorithms were covered in this framework such as XGBoost, the Distributed Random Forest (DRF), the Gradient Boosting Machine (GBM), Generalized Linear Models, the Deep Neural Network, and StackedEnsemble. In this study, the DRF, XGBoost, the GBM, and Generalized Linear Models were adopted. The Deep Neural Network and StackedEnsemble algorithms were excluded in this study because the effectiveness of the feature values could not be calculated in these algorithms.

Third, a random grid search algorithm with H2O AutoML was conducted to tune the hyperparameters in each algorithm, excluding the Random Forest and Extremely Randomized Trees, since the current version of this search algorithm was not supported by these algorithms. In this regard, since each algorithm included several hyperparameters that could not be calculated based on the provided data, inputting appropriate values was required to calculate the algorithms. In this algorithm, combinations of potential values were randomly chosen and inputted in each hyperparameter until a model converged at an optimal value in the performance index, e.g., the mean error rate, the area under the curve (AUC), and F-measures. In this framework, the AUC was set as the performance index, while the maximized value of the AUC was the optimal value. Moreover, the hyperparameters were automatedly tuned, while 5-fold cross validation was conducted for each model.

Finally, the effectiveness of the feature value was calculated. In the tree family algorithm (i.e., the DRF, XGBoost, and the GBM), similar to Gini importance, the magnitude of the values calculated by this framework’s approach represents the contributions to the reduction in squared error for each tree node during the process of dividing trees. In the non-tree-family algorithms, the regression coefficients were calculated. The performance of the algorithms was evaluated with major indices such as the AUC, the area under the precision-recall curve (AUC-PR), and the logarithmic loss metric (logloss). Both the AUC and AUC-PR are performance indices for binary classification problems. Specifically, the AUC ranges from 0 to 1, in which an AUC of “1” means perfect classification, an AUC of “0.5” means chance level, and an AUC of “0” means too-poor performance. As for the AUC-PR, it is appropriate for highly imbalanced data. Unlike the AUC, the AUC-PR focuses on evaluating the true positive, the false positive, and the false negative. Similar to the AUC, an ACU-PR of “1” means perfect classification, an AUC-PR of “0.5” means chance level, and an AUC-PR of “0” means too-poor performance. Moreover, the logloss can be used for both binary and multiclass classification problems. In this regard, the logloss value represents the closeness to the targeted values in the dependent variables. For example, a logloss of “0” means a perfect classifier. In other words, the smaller the logloss, the better the performance classifiers.

## 3. Results

### 3.1. Results of the ALE

**Figure 4:**
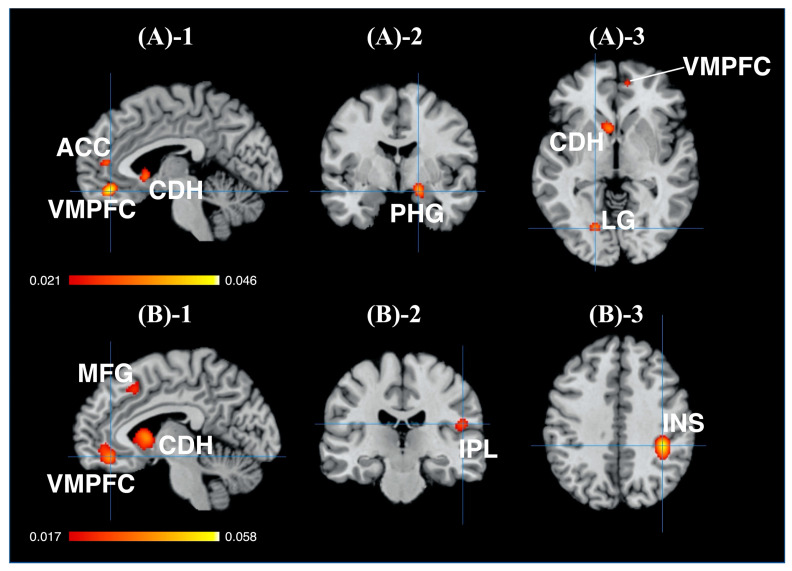
Results of ALE **(A)-1** sagittal view of brand equity-related brain regions, crosshair = (−4, 42, −16); **(A)-2** coronal view of brand equity-related brain regions, crosshair = (18, −4, 16); **(A)-3** axial view of brand equity-related brain regions, crosshair = (−18, −74, −4); **(B)-1** sagittal view of unbranded object-related brain regions, crosshair = (−6, 40, −14); **(B)-2** coronal view of unbranded object-related brain regions, crosshair = (38, −36, 38); **(B)-3** axial view of unbranded object-related brain regions, crosshair = (52, −24, 18). Abbreviations: ALE: activation likelihood estimation; ACC: anterior cingulate cortex; VMPFC: ventromedial prefrontal cortex; CDH: caudate head; PHG: parahippocampal gyrus; LG: lingual gyrus; MFG: middle frontal gyrus; IPL: inferior parietal lobule; INS: insula.

In this study, the activation foci associated with the branded- and unbranded-related regions were revealed. For the branded objects, the activations in the five clusters significantly converged (see Figure 4A and Table 2a). Additionally, the rostral anterior cingulate cortex (rACC, BA32, ventral MPFC [VMPFC]), the medial frontal gyrus (BA10), the parahippocampal gyrus (the entorhinal cortex <BA28>, HP), the caudate head (the anterior part of VS), the posterior cingulate cortex <PCC> (the retrosplenial cortex <RSC>; BA29, BA30), and the lingual gyrus (LG) were observed.

For the unbranded objects, the activations in the six clusters were significantly converged (see Figure 4B and Table 2b). Moreover, the rostral anterior cingulate cortex (rACC, BA32, ventral MPFC [VMPFC]), the medial frontal gyrus (BA24, BA32), the caudate head (the anterior part of the ventral striatum [VS]), the caudate body, the insula (BA13), the IFG (BA40), and the medial frontal gyrus (BA6) were observed.

Based on the results, the anterior part of the MPFC (BA10), the parahippocampal gyrus (PHG) regions (including the hippocampus), the LG, and the PCC were characteristic brain regions, compared with the unbranded-related brain regions. Conversely, both the insula (BA13) and the IFG (BA40) were unique brain regions, compared with the brand equity-related brain regions. Furthermore, both the VMPFC and VS were seemingly overlapped between the brand equity-related brain regions and the unbranded objects-related brain regions.

### 3.2. Results of the Chi-Square Test

**Figure 5:**
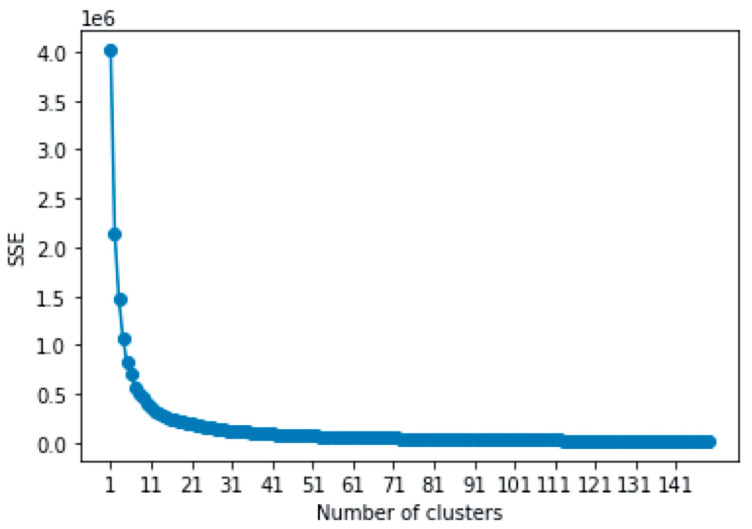
Elbow plot.

**Figure 6:**
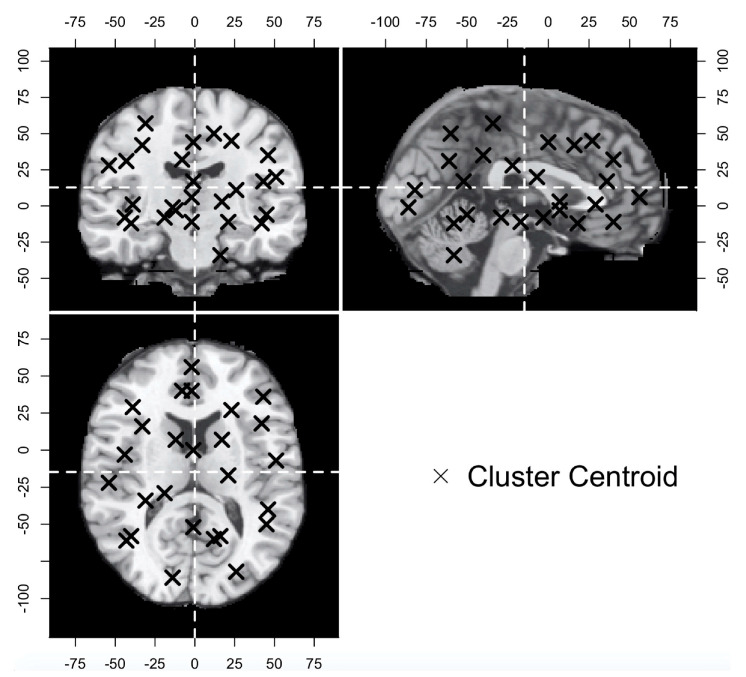
Results of k-means clustering. top left = coronal view; top right = sagittal view; lower right = axial view.

In order to categorize the foci, the k-means algorithm was performed on the initial dataset (see Figure 1 and Table 1). Overall, 26 clusters were determined as the optimal number of clusters by the elbow method. The results of this method are shown in Figure 5 and Supplementary Table S2. Interestingly, the skew of the elbow plot seemed to flatten beyond 20 clusters. As the index of the sum of squared error reached 1/10 in the 24th cluster, The 24th to 26th clusters were chosen as the optimal range (see Supplemental Table S2). Additionally, 26 clusters were adopted as the optimal number of clusters because it was the upper threshold value in the range. The centroid of each cluster is shown in Table 3 and Figure 6. Using the 26 clusters, a chi-square test was conducted. Moreover, the following hypotheses are presented, with H1 representing the null hypothesis and H2 representing the alternative hypothesis:

H1: The cluster ID and the flag are independent;

H2: The cluster ID and the flag are not independent.

The results revealed significant differences between the branded flag and unbranded flag (X^2^ (25) = 45.277, *p* = 0.008). Since H1 was discarded, a residual analysis was conducted to determine the characters of each cluster. Based on the results of the residual analysis shown in Table 4, a significant difference was observed in Clusters 8, 9, 15, 20, and 22. The branded flag also included dominant proportions in Clusters 9, 15, and 20. The findings also indicated that the brain regions belonging to Clusters 9, 15, and 20 can be associated with brand equity-related mental processes. Moreover, the centroid of each cluster corresponded to the PHG (Cluster 9) and the LG (Cluster 15, 20). Conversely, the unbranded flag included dominant proportions in Clusters 8 and 22. The findings also showed that the brain regions belonging to Clusters 8 and 22 were significantly involved in the unbranded objects-related mental processes. As for the centroid of each cluster, it corresponded to the inferior parietal lobule (<IPL>, Cluster 8) and the angular gyrus (Cluster 22), respectively. The other clusters (i.e., Clusters 0–7, 10–16, 17–19, 21, 23–25) were not significantly different.

Finally, the overlapped brain regions were determined by investigating the clusters that did not significantly differ. Since there was almost the same rate between the branded flag and unbranded flag within each cluster (see Table 4), the clusters with a *p*-value higher than 0.45 were determined to be overlapped brain regions. In this regard, Clusters 1, 2, 6, 7, 10, 11, 13, 14, and 21 met this criterion. In Table 4, the corresponding brain regions to the centroid of each cluster are shown. Based on the results, the brain regions in these clusters were more involved in the mental processes of the brand equity-related brain regions and the unbranded objects-related brain regions, compared with the other clusters. Although significant differences were not observed in the other clusters (i.e., Clusters 0, 3–5, 12, 16–19, 23–25), it was presumed that these clusters may have such tendencies such as either brand equity-related brain regions or unbranded-related brain regions, based on the rate between the branded flag and unbranded flag within each cluster.

### 3.3. Results of Machine Learning

The descriptive statistics of feature engineering are shown in Table 5. The data, in which the foci included in the clusters of the overlapped brain regions were eliminated, was used for machine learning. In this case, the XRT algorithm had the best performance. However, the XGBoost algorithm was adopted for analysis because the importance of each feature value could not be calculated in the XRT algorithm. The tuned hyperparameters are listed in Supplementary Table S3, while the performance indices are shown in Table 6. Both the AUC and AUC-PR indicated the chance level. Although the logloss was over 0.5, the results demonstrated that the model can be used to evaluate the predictability of chance level. Additionally, feature importance is shown in Table 7. In this regard, Cluster 9 (the brain regions of the cluster centroid; the right PHG), Cluster 15 (the brain regions of the cluster centroid; the left LG), and Cluster 20 (the brain regions of the cluster centroid; the right LG) had stronger influences on the dependent variables, compared with the other clusters. Considering the proportions of the flags in each cluster, Clusters 9, 15, and 20 had influences on the branded flags because of the dominant proportion in such flags. Meanwhile, Cluster 25 had an influence on the unbranded flags because of the dominant proportion in such flags. In particular, Cluster 9 had the strongest influence on the dependent variables and the highest effectiveness for identifying the branded flags. Thus, the brain regions around the PHG had the strongest influences determining whether objects have brand equity.

## 4. Discussion

Although the characteristic brain regions were not observed regarding the unbranded-related brain regions through the three assessments (i.e., the ALE method, the statistical hypothesis test, and machine learning), this study revealed that the brain regions around both the PHG and the left LG were characteristic brain regions to brand equity and anatomically close to one another. In the right LG, two assessment methods (the statistical hypothesis test and machine learning) were passed. Accordingly, the PHG and LG can be thought of as watershed brain regions for distinguishing mental processes of branded and unbranded objects. Therefore, when metabolic alternations in these regions are observed in a magnetic resonance spectroscopy (MRS) research hereafter, it will imply that the clustered brain regions around both the PHG and LG can be biomarkers for whether brand equity has been established in consumers’ minds. Specifically, the PHG, which corresponds to the centroid of Cluster 9, is associated with recognition [45], episodic memory [46,47,48], and visual and spatial scene processing and navigation [49,50]. In the function of recognition, the anterior part of the PHG is engaged in familiarity-based recognition [51,52]. Meanwhile, the posterior part of the PHG is engaged in recollection-based recognition [53,54]. During recollection, the activations of both the PHG and the posterior parts of the PHG (or single activations of hippocampus) were observed in many cases [45]. In addition, the PHG has a tendency to activate in association with various elements, such as memory sources, and remembering targets when engaging functions of episodic memory [46,47,48]. Thus, episodic memory engaged with the PHG can be thought of as “associative memory” or what Aminoff et al. [55] described as “contextual association”. As for the LG, it is associated with mental imagery [56], visual and spatial scene processing and navigation [49], episodic memory [57], divergent thinking [58], predictive inferences [59], and recognition [60]. These functions, in which the LG is associated in elements of visual processing are required. For example, when generating predictive inferences or performing divergent thinking, visual images must be internally generated. Moreover, the LG also plays a crucial role in language processing, such as in visual recognition of words [61,62] and semantic processing of words [63,64,65]. According to Zhang et al. [65], the LG is involved in language processing and supramodal organization in patients who are not congenitally blind but lost their sight in their early teens. Musz and Thompson [66] demonstrated that the LG plays a role in the semantic hub across the modalities of words. Thus, considering that consumers may recognize a brand as a type of word, the LG is believed to serve as a link connecting modalities and meanings of a brand. Interestingly, these regions are associated with the default mode network (DMN) [67,68]. The PHG is the core region of the DMN [69], and the LG has functional connection with brain regions constituting the DMN [68]. Given that the DMN is engaged in self-referential processing (e.g., episodic memory, autobiographical memory) [67] and associative memory-based autopilot behavior [70], mental processing of branded objects can be thought of as automated mental processes based on associative memories and effortless decision-making based on these mental processes.

Meanwhile, as described earlier, consistent results from the three assessments (i.e., the ALE method, statistical hypothesis test, and machine learning) were not observed in unbranded objects relative to that in branded ones. However, the IPL (BA40) was commonly observed as characteristic brain regions via two assessments (the ALE method and statistical hypothesis test). The IPL associates with calculation [71,72] and decision-making under uncertainty [73,74,75]. Interestingly, connections between the IPL and insula were recorded in metacognition under uncertainty [75]. The insula plays a crucial role in monitoring situations when decision-making under uncertainty. The IPL is involved in controlling and managing mental resources for problem solving under uncertainty. The insula was the brain region revealed in the assessment by the ALE method. In the consumer contexts, the insula detects and evaluates the social risks in purchase decision-making [23]. Besides these regions, the medial frontal gyrus was revealed by the ALE method. The machine learning approach demonstrated that the superior frontal gyrus (Cluster 25) has an influence on unbranded flags. These brain regions are so close that they are placed in a dorsal and medial part of the prefrontal cortex (hereafter, the dorsomedial prefrontal cortex). The dorsomedial prefrontal cortex (DMPFC) is associated with action control, conflict monitoring [76,77], and decision uncertainty [78]. The DMPFC performs these cognitive control-related functions by connecting with the executive control network [78,79]. Additionally, the DMPFC associates with the DMN and is involved in social cognition through a connection with brain regions of the DMN [69]. The DMPFC plays a role in inferring others’ thoughts in complex social relationships [80]. In this way, this region is engaged in organizing and adjusting information to solve problems in complex situations, such as a preference on options with equal values, and unstable situations [81]. Thus, in mental processes of unbranded objects, cognitive control and deliberative aspects may be dominated to handle unknown objects, such as unbranded products and services. In other words, it can be presumed that consumers carefully behave while purchasing unbranded objects.

**Figure 7:**
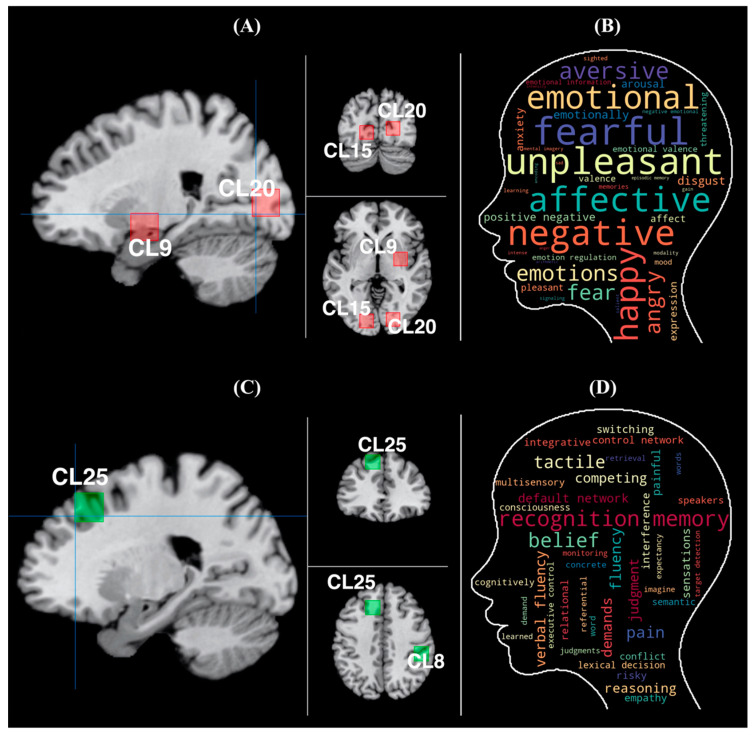
ROI of watershed brain regions and result of decoding study. (**A**) The watershed brain regions for distinguishing branded objects with brand equity and unbranded objects without brand equity; red squares represent ROIs, crosshairs = (22, −79, −3); left = sagittal view; top right = coronal view; lower right = axial view. The brain region of centroid in CL9 is the PHG; the brain region of centroid in CL15 and 20 is the LG. (**B**) The result of decoding study in branded objects via the cognitive decoding function in Neurosynth. (**C**) Clusters with influences on mental processes of unbranded objects; green squires represent ROIs, crosshairs = (−21, 34, 40); left = sagittal view; top right = coronal view; lower right = axial view. The brain region of centroid in CL8 is the IPL (BA40). The brain region of centroid in CL25 is the SFG (BA8, DLPFC). (**D**) The result of the decoding study in unbranded objects via the cognitive decoding function in Neurosynth. Abbreviations: ROI: region of interest; CL: cluster; PHG: parahippocampal gyrus; LG: lingual gyrus; IPL: inferior parietal lobule; SFG: superior frontal gyrus; DLPFC: dorsolateral prefrontal cortex.

Cognitive decoding in Neurosynth (https://neurosynth.org/, accessed on 7 September 2021) was also conducted to more rigorously decode the functions of these clustered brain regions. The decoding analysis was performed for the results of branded objects and unbranded objects. Additionally, the region of interest (ROI) was determined via the Mango software (Version 4.1; http://ric.uthscsa.edu/mango/, accessed on 1 April 2021). In this regard, three ROIs (Cluster 9, 15, and 20) were established, and the shape of each ROI was set as a cube in branded objects. In unbranded objects, two ROIs (Cluster 8, and 25) were established. The length, width, and height in each cube were determined in accordance with the standard deviations of the coordinates in each cluster. Concretely, each measurement of the cube was set at 18 mm. The calculation procedures are as follows. First, the standard deviations (1 sigma) of each coordinate (x, y and z) in each cluster (Clusters 8, 9, 15, 20, and 25) were calculated. Second, the maximum and minimum values of the coordinates in each cluster were determined. For example, in Cluster 9, the maximum value of the x coordinate was determined by calculating 30 (x; centroid of Cluster 9) plus 13 (1 sigma of the x coordinate), while the minimum value of the x coordinate was determined by calculating 30 (x; centroid of Cluster 9) minus 13 (1 sigma of the x coordinate). As for the ranges of the ROI of Cluster 9, the x coordinate ranged from 17 to 43, the y coordinate ranged from −16 to 6, and the z coordinate ranged from −22 to −1. Regarding the ranges of the ROI of Cluster 15, the x coordinate ranged from −24 to −6, the y coordinate ranged from −96 to −77, and the z coordinate ranged from −11 to 11. Regarding the ranges of the ROI of Cluster 20, the x coordinate ranged from 10 to 31, the y coordinate ranged from −94 to −77, and the z coordinate ranged from −4 to 15. Third, each measurement of the cube was adjusted in accordance with these ranges calculated in the second step using the Colin27-T1 template in the Mango software. It was determined that 18 mm was appropriate for the measurement of the cube. Finally, these three ROIs were united into a single ROI (see Figure 7A) in branded objects. Similarly, the two ROIs were united into a single ROI (see Figure 7C) in unbranded objects. After determining the ROI, they were registered in the Neurovault database (https://neurovault.org, accessed on 1 April 2021) for decoding. Subsequently, cognitive decoding was performed for the ROI through the Neurovault database, which is internally connected with Neurosynth. The results of the decoding are shown in Table 8 and Figure 7B,D. In this case, we adopted the top 40 terms, excluding both anatomical terms, disease and experimental task-related terms. The word cloud was created using Python. The higher the correlation values a term had, the larger the font size was set, and vice versa. Accordingly, the font size in the word cloud of branded objects is larger than that of unbranded objects as correlation values in decoded results of branded objects are relatively larger than those in decoded results of unbranded objects. The colors were randomly allocated to each term. In branded objects, the results show that both memory- and emotion-related terms are primarily dominant. Especially, emotion-related terms were ranked as the top 10 terms. This indicates that the emotional episodic memories of objects in consumers’ minds play a crucial role in differentiating between branded objects and unbranded objects. In contrast, in unbranded objects, many executive control-related terms (e.g., “competing”, “judgment”, “reasoning”, “switching”, “control network”, “conflict”, “executive control”, “cognitively”, and “monitoring”) were ranked. Besides this term category, language-related terms (“fluency”, “verbal fluency”, “lexical decision”) and social cognition related terms (“pain”, “default network”, “empathy”) were ranked. Although the decoded terms of unbranded object-related brain regions were not converged into specific categories as were the results of brain regions related to branded objects, the executive control-related terms were characteristic in the decoded results of unbranded objects’ related brain regions.

Overall, the findings of this study are consistent with previous theoretical and empirical brand equity studies. Specifically, the emotional and positive experiences of consumers influence their attitudes toward brands [82], and vice versa. Similarly, it has been revealed that emotional aspects influence value-based decision-making in neuroeconomics and neurofinance studies [83]. These emotional experiences are stored in consumers’ minds along with multimodal sensory information [84]. In addition, the link between emotional episodes and brands help form brand associations, which is one of the crucial elements in brand equity [2]. Hence, a strong brand association is created by episodic memories that are based on emotional experiences [82,85]. In collaboration, this study indicates that the PHG may be involved in emotional aspects of brand associations and the LG may function as a semantic hub connecting various multimodal elements of brand associations. Meanwhile, given that terms related to the executive control network were decoded in analysis of the IPL and the DMPFC, it is presumed that making decisions about unbranded objects may be effortfully executed based on rational mental processes. Therefore, regarding mental processes of branded objects, emotional aspects may be relatively dominant in decision-making. In contrast, cognitive and deliberative aspects may be relatively dominant in mental processes of unbranded objects.

The results of this study also provide useful implications for practitioners. First, when launching a new brand, managers should prioritize the creation of emotional brand associations, aside from other marketing practices. In this regard, they should carefully observe the emotional brand associations and related scores like “familiarity”, in addition to other indices, for tracking brand equity and managing an established brand. Second, when conducting qualitative research, such as in-depth interviews and focus groups, researchers should focus on eliciting emotional episodes on a brand from consumers. In this case, episodes that are visually vivid, spatially concrete, and positively presented can be core factors that strengthen brand associations.

Although the present study provided useful findings to both academicians and practitioners, there are several limitations that should be noted. First, the analyses were conducted using data with stimuli from B2C products and services. In other words, the data in this study included cross product and services data among B2C categories. Depending on these categories, it is possible that different results can be obtained when using data that focus on a specific product/category. In unbranded objects, inconsistent results among the three assessments may be induced owing to these reasons. Further, research on a specific product/category is required in near future. In addition, controlling the attributes and facets in both branded and unbranded objects will be required to overcome the inconsistencies of results for unbranded objects during analysis. Second, the analyses were conducted without considering the heterogeneous sample profiles such as sex, age, occupations, personalities, attitudes toward a brand, and brand usages. In marketing, segmented groups of consumers play a crucial role in setting a strategy and evaluating an outcome. However, in this study, both demographic and psychographic factors were not considered in the analyses. Consequently, it is possible that different results can be derived from these factors. Finally, regarding the analysis by machine learning, it is possible that the performance of the model was improved by conducting the more precise feature engineering. For example, the latter approach added other variables such as raw coordinate data, a task factor, and product categories. Therefore, the results of this study should be carefully interpreted before drawing any conclusions. In this way, although the study has several limitations with this approach, this is the first study in which the watershed brain regions between the branded and unbranded objects were comprehensively revealed based on the enormous brain regions that activated imaging data. However, additional work is required for more precisely identifying a neural mechanism of brand equity and mental processes in it.

## 5. Conclusions

This study identified the unique brain regions related to brand equity and assessed the mental processes derived from these regions. For this purpose, three analysis methods (i.e., the qualitative meta-analysis approach, chi-square tests, and machine learning) were conducted. In total, 65 studies (1412 foci) investigating branded objects with brand equity and unbranded objects without brand equity were examined, while the neural systems involved for these two brain regions were contrasted. Based on the findings, the brain regions around the PHG and LG were the watershed nodes for distinguishing branded objects with brand equity and unbranded objects without brand equity. This study revealed that both the PHG and LG can be involved in a brand association. In particular, the PHG might be engaged in emotional episodic elements of a brand association. Meanwhile, the LG might play a crucial role in the semantic hub on a brand association via word processing. This study indicated that mental processes of branded objects may be automatic information processing based on emotional associative memories derived from these regions, while unbranded objects’ related mental processes may be deliberative and cognitive mental processes.

## Figures and Tables

**Table 1:** Descriptive statistics.

Database	Variables	Variable Type	N	Mean	SD	Median	Min	Max
ALL	x	Numerical	1412	0.43	32.42	−2	−69.17	70.58
	y	Numerical	1412	−10.88	41.99	−6	−105.07	72.9
	z	Numerical	1412	11.39	24.5	8	−52	75.36
	Flag	Categorical	1412	-	-	-	-	-
Branded	x	Numerical	679	1.74	32.04	0	−69.17	70.58
	y	Numerical	679	−13.18	43.3	−8	−105.07	72.56
	z	Numerical	679	9.57	23.7	6	−48	75.36
	Flag	Categorical	679	-	-	-	-	-
Unbranded	x	Numerical	733	−0.78	32.74	−3	−66	69
	y	Numerical	733	−8.74	40.66	−3	−105	72.9
	z	Numerical	733	13.08	25.12	11	−52	74
	Flag	Categorical	733	-	-	-	-	-

**Table 2:** Results of ALE.

(a) Brand Equity−Related Brain Regions								
Cluster #	Side	Brain Region	BA	Peak Voxel Coordinates (MNI)			ALE Values	Cluster Size (mm^3^)
				x	y	z		
1	L	ACC (rostral region/VMPFC)	32	−4	42	−16	0.046	6368
	L	ACC (MPFC)	32	−4	44	8	0.030	
	R	ACC (rostral region/MPFC)	32	10	50	−6	0.027	
	L	Medial Frontal Gyrus (MPFC)	10	−10	52	10	0.023	
	L	Medial Frontal Gyrus (MPFC)	10	0	58	6	0.021	
2	R	PHG (entorhinal cortex)	28	18	−4	−16	0.036	2216
	R	PHG (hippocampus)	−	30	−18	−18	0.020	
3	L	Caudate Head (VS)	−	−6	12	−4	0.034	1936
4	R	PCC (retosplenial region)	30	6	−52	16	0.026	1064
	L	PCC (retosplenial region)	30	−6	−58	12	0.020	
	L	PCC (retosplenial region)	29	−4	−50	14	0.019	
5	L	Lingual Gyrus	18	−18	−74	−4	0.033	1032
	L	Lingual Gyrus	18	−6	−78	−2	0.019	
**(b) Unbranded Objects−Related Brain Regions**								
**Cluster #**	**Side**	**Brain Region**	**BA**	**Peak Voxel Coordinates(MNI)**			**ALE** **Values**	**Cluster Size (mm^3^)**
				**x**	**y**	**z**		
1	L	ACC (rostral region/VMPFC)	32	−6	40	−14	0.0392	3912
	L	ACC (MPFC)	24	−2	36	4	0.0299	
	R	ACC (MPFC)	32	4	44	2	0.0205	
2	L	Caudate Head (VS)	−	−10	10	0	0.0448	3624
3	R	Inferior Parietal Lobule	40	38	−36	38	0.0576	2560
4	R	Caudate Head (VS)	−	12	14	−8	0.0300	1608
	R	Caudate Body	−	10	14	6	0.0254	
5	R	Insula	13	52	−24	18	0.0286	1024
6	L	Medial Frontal Gyrus	6	−4	20	44	0.0258	1000

BA: Brodmann Area; MNI: Montreal Neurological Institute; ALE: activation likelihood estimation; L: left; R: right; ACC: anterior cingulate cortex; VMPFC: ventromedial prefrontal cortex; MPFC: medial prefrontal cortex; PHG: Parahippocampal Gyrus; VS: ventral striatum; PCC: posterior cingulate cortex.

**Table 3:** Centroids in each cluster.

Cluster_ID	Coordinates (MNI)			L/R	Brain Regions
	x	y	z		
cl_0	15	−59	−36	R	Pyramis
cl_1	−1	44	−10	L	Medial Frontal Gyrus (VMPFC)
cl_2	−40	9	35	L	Middle Frontal Gyrus (BA6)
cl_3	0	−54	11	I	Posterior Cingulate
cl_4	48	8	24	R	Inferior Frontal Gyrus
cl_5	−2	10	3	L	Lateral Ventricle
cl_6	−38	−57	−12	L	Fusiform Gyrus (BA37)
cl_7	−47	−4	−4	L	Superior Temporal Gyrus (BA22)
cl_8	48	−32	32	R	Inferior Parietal Lobule (BA40)
cl_9	30	−5	−11	R	PHG
cl_10	24	37	36	R	Superior Frontal Gyrus
cl_11	−43	−60	29	L	Middle Temporal Gyrus
cl_12	−39	31	2	L	Inferior Frontal Gyrus
cl_13	41	−48	−8	R	Sub−Gyral
cl_14	−25	6	−16	L	Subcallosal Gyrus
cl_15	−15	−87	0	L	Lingual Gyrus (BA17)
cl_16	4	−57	47	R	Precuneus (BA7)
cl_17	41	34	0	R	Inferior Frontal Gyrus
cl_18	−54	−25	27	L	Inferior Parietal Lobule
cl_19	−12	−23	−9	L	Midbrain
cl_20	20	−85	5	R	Lingual Gyrus (BA17)
cl_21	5	−8	40	R	Middle Cingulate Gyrus (BA24)
cl_22	40	−64	30	R	Angular Gyrus
cl_23	−5	53	16	L	Medial Frontal Gyrus (BA9)
cl_24	−31	−33	57	L	Postcentral Gyrus
cl_25	−13	26	46	L	Superior Frontal Gyrus (BA8)

BA: Brodmann Area; MNI: Montreal Neurological Institute; L: left; R: right; VMPFC: ventromedial prefrontal cortex; PHG: parahippocampal gyrus.

**Table 4:** Results of chi-square test.

Cluster_ID	Contingency Table		Adjusted Residual		*p*-Value	
	Branded	Unbranded	Branded	Unbranded	Branded	Unbranded
cl_0	17	25	−1.0023	1.0023	0.3162	0.3162
cl_1	37	46	−0.6596	0.6596	0.5095	0.5095
cl_2	19	25	−0.6617	0.6617	0.5081	0.5081
cl_3	29	23	1.1296	−1.1296	0.2586	0.2586
cl_4	29	22	1.2775	−1.2775	0.2014	0.2014
cl_5	36	52	−1.3919	1.3919	0.164	0.164
cl_6	29	31	0.0389	−0.0389	0.969	0.969
cl_7	23	26	−0.1639	0.1639	0.8698	0.8698
cl_8	29	50	−2.0834	2.0834	0.0372 **	0.0372 **
cl_9	54	29	3.1900	−3.1900	0.0014 ***	0.0014 ***
cl_10	20	25	−0.4972	0.4972	0.6191	0.6191
cl_11	21	22	0.0999	−0.0999	0.9204	0.9204
cl_12	26	37	−1.1081	1.1081	0.2678	0.2678
cl_13	28	25	0.7044	−0.7044	0.4812	0.4812
cl_14	19	23	−0.3753	0.3753	0.7075	0.7075
cl_15	34	15	3.0373	−3.0373	0.0024 ***	0.0024 ***
cl_16	19	30	−1.3279	1.3279	0.1842	0.1842
cl_17	28	39	−1.0570	1.0570	0.2905	0.2905
cl_18	14	24	−1.4065	1.4065	0.1596	0.1596
cl_19	24	16	1.5297	−1.5297	0.1261	0.1261
cl_20	29	7	3.9497	−3.9497	0.0001 ***	0.0001 ***
cl_21	21	27	−0.6120	0.6120	0.5405	0.5405
cl_22	12	24	−1.7949	1.7949	0.0727 *	0.0727 *
cl_23	43	34	1.4010	−1.4010	0.1612	0.1612
cl_24	10	18	−1.3236	1.3236	0.1856	0.1856
cl_25	29	38	−0.8064	0.8064	0.42	0.42

* *p* < 0.1, ** *p* < 0.05, *** *p* < 0.005. Gray-shaded areas are statistically significant.

**Table 5:** Descriptive statistics (after feature engineering).

Database	Variables	Variable Type	N	Mean	SD	Median	Min	Max
ALL	x	Numerical	945	6.85	30.89	4	−68.67	70.58
	y	Numerical	945	−12.9	42.89	−12	−105.07	72.56
	z	Numerical	945	13.28	24.15	12	−52	75.36
	Flag	Categorical	945	-	-	-	-	-
Branded	x	Numerical	462	7.89	29.7	6	−68.67	70.58
	y	Numerical	462	−15.05	44.53	−13	−105.07	72.56
	z	Numerical	462	10.84	22.96	8.26	−48	75.36
	Flag	Categorical	462	-	-	-	-	-
Unbranded	x	Numerical	483	5.85	31.99	2	−66	69
	y	Numerical	483	−10.84	41.2	−10	−105	66
	z	Numerical	483	15.61	25.04	15	−52	74
	Flag	Categorical	483	-	-	-	-	-

**Table 6:** Performance indices.

Rank	Model_ID	AUC	Logloss	AUC-PR
1	XRT_1_AutoML_20210907_131623	0.5841	0.6804	0.5426
2	XGBoost_1_AutoML_20210907_131623	0.5826	0.6794	0.5449
3	GBM_2_AutoML_20210907_131623	0.5810	0.6813	0.5390
4	XGBoost_3_AutoML_20210907_131623	0.5796	0.6833	0.5375
5	GBM_4_AutoML_20210907_131623	0.5782	0.6814	0.5361
6	DRF_1_AutoML_20210907_131623	0.5774	0.6827	0.5378
7	XGBoost_grid__1_AutoML_20210907_131623_model_1	0.5770	0.6829	0.5383
8	XGBoost_grid__1_AutoML_20210907_131623_model_6	0.5759	0.6810	0.5333
9	GBM_grid__1_AutoML_20210907_131623_model_6	0.5757	0.6809	0.5359
10	GBM_5_AutoML_20210907_131623	0.5757	0.6803	0.5343

AUC: area under the curve; logloss: logarithmic loss metric; AUC-PR: area under the precision-recall.

**Table 7:** Feature importance.

Feature Values	Cluster Centered Brain Regions	Relative_Importance	Scaled_Importance
Cluster_id.cl_9	PHG	29.023	1.000
Cluster_id.cl_15	Lingual Gyrus (BA17)	20.785	0.716
Cluster_id.cl_20	Lingual Gyrus (BA17)	19.454	0.670
Cluster_id.cl_25	Superior Frontal Gyrus (BA8)	15.762	0.543
Cluster_id.cl_16	Precuneus (BA7)	13.721	0.473
Cluster_id.cl_19	Midbrain	12.747	0.439
Cluster_id.cl_5	Lateral Ventricle	11.915	0.411
Cluster_id.cl_17	Inferior Frontal Gyrus	11.477	0.396
Cluster_id.cl_4	Inferior Frontal Gyrus	11.266	0.388
Cluster_id.cl_3	PCC	10.938	0.377

Feature values were sorted by importance values; BA: Brodmann Area; PHG: parahippocampal gyrus; PCC: posterior cingulate cortex.

**Table 8:** Results of decoding study by Neurosynth. Each term was sorted by higher correlation values order.

Branded Objects		Unbranded Objects	
Cognitive Terms	Correlation	Cognitive Terms	Correlation
Fearful	0.129	Recognition memory	0.079
Unpleasant	0.124	Belief	0.068
Negative	0.116	Tactile	0.051
Affective	0.109	Pain	0.043
Emotional	0.101	Fluency	0.036
Happy	0.098	Demands	0.035
Aversive	0.098	Verbal fluency	0.033
Emotions	0.093	Competing	0.03
Angry	0.09	Judgment	0.03
Fear	0.088	Reasoning	0.029
Emotionally	0.08	Default network	0.025
Disgust	0.078	Sensations	0.023
Positive negative	0.077	Painful	0.022
Arousal	0.071	Interference	0.02
Anxiety	0.068	Switching	0.02
Expression	0.065	Integrative	0.019
Affect	0.06	Relational	0.019
Pleasant	0.054	Empathy	0.019
Valence	0.052	Risky	0.019
Threatening	0.049	Control network	0.019
Emotional valence	0.049	Conflict	0.017
Emotion regulation	0.047	Multisensory	0.017
Mood	0.045	Speakers	0.017
Emotional information	0.041	Consciousness	0.016
Memories	0.038	Lexical decision	0.016
Sighted	0.036	Semantic	0.016
Learning	0.029	Cognitively	0.015
Negative emotional	0.029	Executive control	0.014
Modality	0.027	Concrete	0.013
Intense	0.024	Referential	0.013
Signaling	0.023	Word	0.013
Salient	0.022	Demand	0.012
Mental imagery	0.021	Retrieval	0.011
Gain	0.021	Words	0.011
Sad	0.019	Imagine	0.011
Episodic memory	0.018	Learned	0.011
Encoding	0.016	Monitoring	0.011
Intensity	0.013	Expectancy	0.011
Arithmetic	0.013	Judgments	0.011
Anger	0.012	Target detection	0.009

## Data Availability

The datasets in this study are available by request to the corresponding author. The statistical and pattern weight maps are available on the Neurovault repository, under collection 11099 (https://neurovault.org/collections/KGRYTGFI/, accessed on 7 September 2021 and 11539 (https://neurovault.org/collections/WZWOAKWH/, accessed on 25 October 2021).

## Supplementary Materials

The following are available online at https://www.mdpi.com/article/10.3390/brainsci11121619/s1, Table S1(a): Branded studies included in the meta-analysis, Table S1(b): Unbranded studies included in the meta-analysis. Table S2: Convergence of sum of squared error (SSE). Table S3: Results of hyperparameter tuning.
