# Supplementary material for: Watershed Brain Regions for Characterizing Brand Equity-Related Mental Processes
Source: Brain Sci. 2021 Dec 8;11(12):1619. doi: 10.3390/brainsci11121619 (PMC8699238; doi:10.3390/brainsci11121619)
Supplement: Supplementary file 1 [file brainsci-11-01619-s001.zip › brainsci-1470362-supplementary.pdf]

Supplementary Tables

Table S1(a). Branded studies included in the meta-analysis.

Experiment	Subject	Foci	Experiment stimuli	Detailed information
Erk et al.(2002)	12	7	Package images	Cultural objects (cars; sports car/ limousine /small cars)
		2		
		2		
		2		
McClure et al.(2004)	16	7	Brand logos/Carbonated drinks(Coke/Pepsi)	Culturally Familiar Drinks
Deppe et al.(2005a)	21	5	Brand logos	Magazines
Deppe et al.(2005b)	22	16	Package images with brand logos	Beer/Coffee
		2		
		8		
		11		
Schaefer et al.(2006)	13	1	Brand logos	culturally familiar brands（Cars)
Yoon et al.(2006)	19	1	Brand names/Adjectivess	
Plassmann et al.(2007)	22	1	Brand logos(clothing images)	Departmant store
Schaefer & Rotte(2007a)	13	5	Brand logos	Cars
Schaefer & Rotte(2007b)	14	5	Brand logos	Cars
		1		
		5		
		2		
Koeneke et al.(2008)	19	28	Real products	Chochorate bars
Plassmann et al.(2008)	20	8	Wine	Intake
		10		
		12		
		6		
		2		
		5		
		8		
		1		

Klucharev et al. (2008)	18	10	Package images without brand logos	Clothes, cosmetics, packaged food, etc.
	18	7		
	16	4		
	18	4		
Kato et al.(2009)	40	18	Advertising with brand logos	Coke Ad/Pepsi Ad
		8		
		3		
		26		
Schaefer et al.(2011)	12	2	Package images with brand logos	Chochorates
Reimann et al.(2011)	16	1	Musician and title	Listen before experiments
		2		
Casarotto et al.(2012)	15	4	Brand logos	13 differeint product categories(clothing, transport, food, gas, cigarettes, etc.)
		1		
		19		
Esch et al.(2012)	15	2	Brand names and logo	8 strong brands(BMW, Coca Cola, etc) and 8 weak brands(Kia, Yahoo, etc.)and 8 unfamiliar brands
		2		
		4		
		2		
		4		
Murawski et al.(2012)	13	11	Brand logos	Cups with Apple logo
		5		
		2		
	9	1		
	13	1		
	11	4		
	13	5		
Reimann et al.(2012)	16	6	Brand names and logo	Disney, Google, Apple and Starbucks Coffee. Etc.
Grabenhorst et al.(2013)	13	2	Fooods with Taste label/Health label	
		4		
		2		
		2		

		1		
		3		
		4		
Bruce et al.(2014)	17	7	Brand logos	foods(60):pizzahut,kfc,etc./non foods(60):lego,spongebob,windows,etc.
		5		
		4		
Burger and Stice(2014)	9	10	Product with logo	Coke Ad/Non food Ad
	25	12	Product & logo ad	
		37		
		8		
		11		
		4		
Chen et al(2015)	17	23	Brand logo	44 brands listed by the intetrand brand ranking
Enax et al.(2015)	40	9	Food images with FT certified marks	Various food category (chocolate, coffee, rice, etc..)
		5		
		7		
		10		
		4		
		4		
		6		
		3		
		2		
Audrin et al.(2017)	38	20	Items and brand logo images	Luxuarious and Non-luxuarious(scarves, handbags, belts and purses)
		26		
		2		
		2		
Jung et al.(2018)	34	21	Food images with logo (social/conventional enterprises)	Confectionery(i.e., cookies, chocolate, bread, and Korean traditional rice cake)
		15		
		9		
		8		
		11		
		4		

		4		
		25		
		1		
		21		

Abbreviations; FT, fairtrade

Table S1(b). **Unbranded studies included in the meta-analysis.**

Experiment	Subject	Foci	Experiment stimuli	Detailed information
McClure et al.(2004)	15	2	Carbonated drinks(No branded)	
O'Doherty et al.(2006)	13	6	Flavored drinks	
Knutson et al.(2007)	26	14	Product images	
		6		
		18		
Plassmann et al.(2008)	20	1	Wine	Intake
		10		
		2		
Chib et al.(2009)	19	1	money/trinkets/snacks	
		1		
		1		
		1		
Berns et al.(2010)	27	3	Songs(15s clips)	
		4		
		2		
		7		
		2		
Esch(2010)		2	Unfamiliar brand	
Tusche et al.(2010)		8	Cars(Images)	
		14		
		22		
		2		
		2		
Murawski et al.(2012)	13	1	Product images	

	11	4		
Van der Laan et al.(2012)	20	8	Food packages and products(images)	
		2		
		10		
Creswell et al.(2013)	27	6	Cars	
		2		
		8		
		4		
Kang and Camerer(2013)	27	17	Food images	
Lee et al.(2013)	23	3	Food images	
Burger and Stice.(2014)	9	11	Milkshake	
He Q et al.(2014)	30	5	Food images	High-calorie food(chocolate bars, cookies, ice cream, and potato chips) /Low-calorie food(celery, broccoli, and carrots.)
		1		
		7		
		1		
Lighthall et al.(2014)	42	4	Product images	14 product categories(clothing, kitchen appliances, watch, etc.)
		3		
		6		
		3		
		1		
Yokoyama et al.(2014)	30	3	Product images	T-shirts
		2		
Giuliani and Pfeifer(2015)	60	16	Food images	Energy density(ED) and low ED foods(e.g., chocolate, cookies, carrots, corn etc..)
		12		
		3		
		16		
		15		
		2		
		8		
		9		

Schaefer et al.(2015)	10	5	Product images	3 product categories(cameras, cars, or cosmetics)
Petit et al.(2016)	22	7	Food images	Various food categories(e.g., junk food, healthy snacks)
		2		
		5		
		6		
		17		
		4		
Stuke et al(2016)	38	5	Drink images	Alcohol dirnks/Non-alcohol drinks
		4		
Waskow et al(2016)	25	3	Music albums	Genres: rock, metal, hip hop, country/folk, indie, and pop
		1		
De Martino et al(2017)	18	1	Product images	210 pictures of products from the retail website Amazon
		1		
		1		
		1		
Dal Mas et al.(2017)	30	2	Musician and title	Music and Songs
		11		
Goodman et al.(2017)	10	7	Product images	Generic product<br>(Various product categories；bikes, blu-ray players, mugs, etc.)
		5		
		1		
		4		
		4		
Tapp et al.(2017)	8	4	Food images	Beef steaks
		7		
		7		
		5		
		8		
Hege et al.(2018)	23	3	Food images	Meals
		1		
		1		
		1		

Francisco(2018)		12		
Liu et al.(2018)	25	6	Food and products images	28 types of food and 28 objects (bread/chocolate/mug/toothbrush etc..)
		4		
		4		
		2		
Ceravolo et al.(2019)	16	3	Video clips(Payment methods)	cash,credit card,smartphone
		2		
		2		
		2		
		2		
		2		
		2		
		2		
		2		
		3		
Ceravolo et al.(2019)		3		
Huijsmans et al.(2019)	47	1	Food images	144 different supermarket food items
		1		
Kageyama et al(2019)	30	4	Product images	cars, massage chairs, and apartments
		3		
Masterson TD et al(2019)	41	10	Food images	Energy dense foods (e.g., Low:vegetables and fruit/High: candy and ice-cream)
		6		
		4		
Muñoz-Leiva et al.(2019)	24	7	Food images	Meals(restaurant dishes)
		5		
		13		
Motoki et al.(2019)	27	3	Product images	Books
		10		
		1		
		1		
Setton et al(2019)	16	26	Food images(package)	Snack items

		43		
		9		
		26		
		13		
		3		
Tijssen et al.(2019)	34	3	Package images	Daily drinks
		7		
		1		
Dubey(2020)	24	7	Movies(social and nonsocial stimuli)	
		10		
		5		

Table S2. Convergence of sum of squared error (SSE)

**Number of clusters**	**Sum of squared error**	**Change**
1	4818454	100
2	2957686	61
3	2314769	48
4	1895791	39
5	1651521	34
6	1433509	30
7	1250573	26
8	1131221	23
9	1035885	21
10	954674	20
11	893590	19
12	840672	17
13	800432	17
14	754206	16
15	720331	15
16	690008	14
17	652860	14
18	616430	13
19	594523	12
20	570957	12
21	552250	11
22	531716	11
23	506857	11
24	497241	10
25	481176	10
26	465804	10

27	450966	9
28	439433	9
29	427111	9
30	413699	9
31	400233	8
32	394927	8
33	387283	8
34	378668	8
35	369896	8
36	360019	7
37	355298	7
38	347697	7
39	334542	7
40	330538	7
41	327162	7
42	322552	7
43	317067	7
44	313363	7
45	305557	6
46	299317	6
47	297043	6
48	289486	6
49	285231	6
50	280468	6
51	273000	6
52	270646	6
53	265304	6
54	262975	5
55	257883	5
56	256234	5
57	253429	5
58	250942	5
59	247597	5
60	243698	5
61	240089	5
62	236650	5
63	234768	5
64	232320	5
65	229655	5
66	224782	5
67	222176	5
68	220720	5
69	218083	5
70	215566	4
71	213670	4
72	210964	4

73	208391	4
74	204162	4
75	203477	4
76	201939	4
77	199121	4
78	197310	4
79	196194	4
80	194820	4
81	191048	4
82	189070	4
83	187414	4
84	185478	4
85	184122	4
86	182802	4
87	180683	4
88	178953	4
89	177990	4
90	176631	4
91	174701	4
92	174062	4
93	171986	4
94	170293	4
95	168579	3
96	167863	3
97	165368	3
98	163140	3
99	161348	3
100	160288	3
101	159567	3
102	157959	3
103	157318	3
104	156451	3
105	155914	3
106	154363	3
107	153740	3
108	152332	3
109	150696	3
110	149240	3
111	148392	3
112	146756	3
113	145113	3
114	143809	3
115	142629	3
116	142207	3
117	141328	3
118	140117	3

119	139318	3
120	137719	3
121	137086	3
122	136014	3
123	135562	3
124	134312	3
125	133293	3
126	132785	3
127	132000	3
128	130930	3
129	129315	3
130	128634	3
131	127690	3
132	126735	3
133	125472	3
134	124728	3
135	123331	3
136	122438	3
137	121103	3
138	120251	2
139	119666	2
140	119055	2
141	118176	2
142	117631	2
143	116966	2
144	116753	2
145	116363	2
146	115804	2
147	114993	2
148	114188	2
149	112107	2
150	111439	2

Change: the index, which is calculated as the basis of the SSE value of the first cluster, represents how much ratios SSEs were reduced against the SSE of first cluster.

Table S3. Results of hyperparameter tuning

Label	Actual_value	Default_value
model_id	XGBoost_1_AutoML_20210907_131623	
stopping_rounds	0	0
stopping_tolerance	0.032530002	0.001
max_runtime_secs	0	0
seed	-8.26687E+18	-1
distribution	bernoulli	AUTO
tweedie_power	1.5	1.5
ntrees	30	50

max_depth	10	6
min_rows	5	1
min_child_weight	5	1
learn_rate	0.3	0.3
eta	0.3	0.3
sample_rate	0.6	1
subsample	0.6	1
col_sample_rate	0.8	1
colsample_bylevel	0.8	1
col_sample_rate_per_tree	0.8	1
colsample_bytree	0.8	1
colsample_bynode	1	1
max_abs_leafnode_pred	0	0
max_delta_step	0	0
score_tree_interval	5	0
min_split_improvement	0	0
gamma	0	0
auc_type	AUTO	AUTO
nthread	-1	-1
max_bins	256	256
max_leaves	0	0
sample_type	uniform	uniform
normalize_type	tree	tree
rate_drop	0	0
one_drop	FALSE	FALSE
skip_drop	0	0
tree_method	exact	auto
grow_policy	depthwise	depthwise
booster	gbtree	gbtree
reg_lambda	1	1
reg_alpha	0	0
dmatrix_type	sparse	auto
